# Distinct characteristics of microglia from neurogenic and non-neurogenic regions of the human brain in patients with Mesial Temporal Lobe Epilepsy

**DOI:** 10.3389/fncel.2022.1047928

**Published:** 2022-11-08

**Authors:** Amy M. Smith, Thomas In-Hyeup Park, Miranda Aalderink, Robyn L. Oldfield, Peter S. Bergin, Edward W. Mee, Richard L. M. Faull, Mike Dragunow

**Affiliations:** ^1^Department of Pharmacology and Clinical Pharmacology, The University of Auckland, Auckland, New Zealand; ^2^Centre for Brain Research, The University of Auckland, Auckland, New Zealand; ^3^LabPLUS, Auckland, New Zealand; ^4^Auckland City Hospital, Auckland, New Zealand; ^5^Department of Anatomy and Medical Imaging, The University of Auckland, Auckland, New Zealand

**Keywords:** microglia, primary human cell culture, M-CSF or CSF1, neurogenic niche, microglial heterogeneity

## Abstract

The study of microglia isolated from adult human brain tissue provides unique insight into the physiology of these brain immune cells and their role in adult human brain disorders. Reports of microglia in post-mortem adult human brain tissue show regional differences in microglial populations, however, these differences have not been fully explored in living microglia. In this study biopsy tissue was obtained from epileptic patients undergoing surgery and consisted of both cortical areas and neurogenic ventricular and hippocampal (Hp) areas. Microglia were concurrently isolated from both regions and compared by immunochemistry. Our initial observation was that a greater number of microglia resulted from isolation and culture of ventricular/Hp tissue than cortical tissue. This was found to be due to a greater proliferative capacity of microglia from ventricular/Hp regions compared to the cortex. Additionally, ventricular/Hp microglia had a greater proliferative response to the microglial mitogen Macrophage Colony-Stimulating Factor (M-CSF). This enhanced response was found to be associated with higher M-CSF receptor expression and higher expression of proteins involved in M-CSF signalling DAP12 and C/EBPβ. Microglia from the ventricular/Hp region also displayed higher expression of the receptor for Insulin-like Growth Factor-1, a molecule with some functional similarity to M-CSF. Compared to microglia isolated from the cortex, ventricular/Hp microglia showed increased HLA-DP, DQ, DR antigen presentation protein expression and a rounded morphology. These findings show that microglia from adult human brain neurogenic regions are more proliferative than cortical microglia and have a distinct protein expression profile. The data present a case for differential microglial phenotype and function in different regions of the adult human brain and suggest that microglia in adult neurogenic regions are “primed” to an activated state by their unique tissue environment.

## Introduction

Microglia are the brain’s primary immune cells. They play important homeostatic roles and can modulate the functions of other brain cells. Microglia actively monitor neuronal synapses (Nimmerjahn et al., 2005; Wake et al., 2009), phagocytose debris (Chan et al., 2001; Simard et al., 2006), and can secrete both supportive and detrimental factors into the extracellular environment (Olah et al., 2011). Microglia communicate with other brain cells *via* cytokines and growth factors and are important cell types during development, in normal physiological states, and during injury and degenerative processes (de Haas et al., 2007). In these different situations, microglia can express specific cell surface receptors, have specific morphology, and produce different soluble molecules (Olah et al., 2011). Thus, microglia have marked phenotypic diversity which is influenced by their microenvironment including other cell types and soluble molecules in their surroundings. An important factor regulating microglial phenotype is the growth factor Macrophage Colony-Stimulating Factor (M-CSF). Development and differentiation of microglia is dependent on M-CSF signalling (Ginhoux et al., 2010) and M-CSF also increases microglial division (Lee et al., 1993; Vidyadaran et al., 2009; Yamamoto et al., 2010).

Normal rodent and human adult brains have regional differences in microglia density and immune protein expression (Mittelbronn et al., 2001; de Haas et al., 2008). For example, it has been demonstrated that white matter contains more microglia than grey matter (Mittelbronn et al., 2001). Furthermore, different brain regions show different microglial responses to ageing (Hart et al., 2012) and different phenotypes *in vitro* (Melief et al., 2012). The diverse nature of these brain immune cells has begun to be appreciated, but it is not fully known to what extent microglia vary in different brain regions and what factors/mechanisms produce these differences.

Two highly specialised areas of the adult brain are the neurogenic regions of the subventricular zone (SVZ) of the lateral ventricles and the dentate gyrus (DG) of the hippocampus. Here neural progenitor cells (NPCs) proliferate throughout life and give rise to new neurons (Eriksson et al., 1998; Curtis et al., 2007). Microglia are present in both the rodent and human SVZ and DG (Curtis et al., 2005; Goings et al., 2006; Hellstrom et al., 2011; Morrens et al., 2012) where they perform many important roles including cytokine production and phagocytosis (Walton et al., 2006; Sierra et al., 2010; Morrens et al., 2012). Investigation of the role of microglia in adult neurogenesis has led to findings of specific microglial characteristics within the SVZ and DG neurogenic regions. Microglia of the mouse SVZ were found to have a higher level of basal activation and proliferation, and greater expression of CD45 and CD11b, than non-neurogenic regions of striatum and corpus callosum (Goings et al., 2006). Following cortical injury, SVZ microglia did not, however, become more activated, despite being closer to the injury than the striatum and corpus callosum where microglia greatly increased activation compared to non-injured brains (Goings et al., 2006). A study of human microglial regional heterogeneity found that SVZ microglia clustered separately from microglia from the cortex, cerebellum or thalamus, and identified a phenotypic subset of microglia in the SVZ that was absent from the temporal and frontal cortex (Böttcher et al., 2019). Further evidence of a special phenotype of microglia in neurogenic regions is the unique proliferative capacity of microglia found in the SVZ of neonatal mice whereby SVZ cultures produced 20-fold greater yields of microglia than corresponding cortical cultures (Marshall et al., 2008; Marshall et al., 2014). However, adult brain cultures gave 10 times fewer microglia (Marshall et al., 2008) and it is unknown whether this unique proliferative capacity of subventricular microglia is true for the adult human brain as several other differences exist between the culture of rodent and human microglia.

The use of primary human microglia is an invaluable tool for neuroscience research (Dragunow, 2008a; Gibbons and Dragunow, 2010; Smith and Dragunow, 2014). Using human biopsy tissue from temporal lobe epilepsy surgeries, the microglia of the hippocampus and of the temporal horn of the lateral ventricle (herein referred to as “ventricular/Hp” microglia) were compared to microglia from the cortical middle temporal gyrus. Major distinctions were found between microglia from these two separate human adult brain regions in terms of proliferation and immune protein expression.

## Materials and methods

### Tissue

Biopsy adult human brain tissue used for this study was obtained from patients undergoing surgery for drug-resistant temporal lobe epilepsy. This research was approved by the Northern Regional Ethics Committee and the University of Auckland Human Participants Ethics Committee and informed consent was obtained from all participants. All biopsy specimens were from temporal lobe epilepsy cases with a high degree of mesial temporal sclerosis (neuropathological grade 3–4, where grade 4 is maximal severity). Due to the nature of the surgery, all candidates had previously been on a range of medications, administered alone or in combination, including lamotrigine, phenytoin, sodium valproate, tegretol, and topiramate. All patients were male and in their third decade. Biopsy tissue with gross signs of sclerosis (i.e., the seizure focus) was removed by a pathologist for pathological examination and the remaining cortical and ventricular/Hp tissue was used for the culture and study of microglia.

### Human glial cell isolation and culture

Microglia were isolated from two regions of adult human brain tissue using a method based on the cell isolation protocols previously optimised in our lab (Gibbons et al., 2011; Park et al., 2012, 2022; Smith et al., 2013a; Rustenhoven et al., 2016). The two brain regions compared were (1) cortical middle temporal gyrus and (2) the neurogenic regions of the hippocampal DG and the overlying SVZ of the lateral ventricle. Tissue of equivalent weights from both regions was processed concurrently by the same method. 1–2 g tissue was washed once in HBSS and visible blood vessels were removed with sterile forceps. The tissue was diced in a sterile petri dish into 1 mm^3^ pieces. The tissue was placed in a 15 ml tube containing 10 ml enzymatic mix [100 U/ml DNase (Invitrogen, Carlsbad, CA, USA) and 2.5 U/ml papain (Worthington, Lakewood, NJ, USA) in Hibernate-A medium (Gibco, Waltham, MA, USA)] for chemical digestion. This was placed on a rotating device in a 37°C incubator for 10 min. The cell/enzyme mix was then removed from the incubator, triturated with a 10 ml pipette, and returned to the incubator for a further 10 min. This cell mix was then diluted with 10 ml DMEM/F12 (Gibco, CA, USA) with 1% B27 (Gibco, CA, USA) and passed through a 70 μm strainer (Bector Dickinson, Franklin Lakes, NJ, USA). The cells that passed through the strainer were centrifuged at 160 × *g* for 10 min and resuspended in 7 ml media [DMEM/F12 with 1% B27, 1% GlutaMAX (Gibco, CA, USA), 1% penicillin-streptomycin (Gibco, CA, USA), 40 ng/ml fibroblast growth factor (FGF; Peprotech, Cranbury, NJ, USA), 40 ng/ml epidermal growth factor (EGF; Peprotech, NJ, USA) and 2 μg/ml heparin (Sigma, St. Louis, MO, USA)] and seeded into a T25 flask. This media has previously been optimised for the survival of neural precursor cells which can also be obtained by this cell isolation method, but which are easily isolated and distinguishable from microglia (Park et al., 2012, 2022; Rustenhoven et al., 2016). On the following day (14–18 h later) the flasks were tapped and non-adherent cells and debris were removed and used for studies of neural precursor cells. The remaining adherent glia were maintained as previously described (Gibbons et al., 2011; Smith et al., 2013a) by adding 7 ml microglial media (DMEM/F12 with 10% FBS and 1% PSG) and culturing for 1–2 weeks. Cells cultured from both brain regions were plated simultaneously into adjacent wells of 96-well plates at 50,000 cells/ml for experimentation.

### Cytokine treatment

Primary human glial cell cultures were treated in 96-well plates. 1 μl cytokine was added to 100 μl media. Cells were treated with 25 ng/ml recombinant human M-CSF (Sigma-Aldrich, MO, USA; in H_2_O) or 1 ng/ml recombinant human IFNy (R&D Systems, Minneapolis, MN, USA; in PBS with 0.1% BSA) at 0 and 48 h. Total time of cytokine treatment was 96 h.

### BrdU proliferation assay

Following 72 h exposure to 25 ng/ml M-CSF, 10 μM BrdU was added to the cells for 24 h. Cells were washed twice with PBS to remove excess BrdU and fixed with 4% paraformaldehyde (PFA) for 15 min at room temperature and processed for immunocytochemistry as previously described (Smith et al., 2013b).

### Immunocytochemistry

Cells were processed for immunocytochemistry as described previously using the specific antibodies detailed in Table 1, which we have previously characterised (Smith et al., 2013b). Controls omitting the primary antibodies gave no staining. Incubation of fixed cells with 0.5 μg Human Fc Block (BD Biosciences, NJ, USA) for 30 min at room temperature prior to the addition of primary antibody produced no significant difference in staining (Supplementary Figure 1).

**TABLE 1:** Antibodies used for immunocytochemistry.

Antibody	Company	Catalogue #	Dilution
Rabbit anti-PU.1	Cell signalling	2258	1:500
Mouse anti-CD45	Abcam	ab8216	1:500
Rabbit anti-CSF-1R	Santa Cruz	Sc-692	1:50
Mouse anti-HLA-DP, DQ, DR	Dako	M0775	1:500
Rabbit anti-DAP12	Santa Cruz	Sc-20783	1:500
Mouse anti-C/EBPβ	Santa Cruz	Sc-7962	1:250
Mouse anti-IGF-1R	Millipore	MAB1120	1:50
Mouse anti-BrdU	Roche	11170376001	1:500
Rabbit anti-Ki67	Dako	A0047	1:500
Goat anti-rabbit IgG Alexa Fluor^®^ 594	Invitrogen	A11012	1:500
Goat anti-mouse IgG Alexa Fluor^®^ 488	Invitrogen	A11001	1:500
Goat anti-mouse IgG Alexa Fluor^®^ 594	Invitrogen	A11005	1:500
Goat anti-rabbit IgG Alexa Fluor^®^ 488	Invitrogen	A11008	1:500

### Quantitative image analysis of cell number, protein expression and microglial morphology

Immunocytochemical and morphological observations were quantified using a Discovery-1 automated fluorescence microscope (Molecular Devices, San Jose, CA, USA) and Metamorph (6.2.6 software, Molecular Devices) image analysis system as previously described (Dragunow, 2008b; Smith et al., 2010). Intensity thresholds were set based on the staining by each antibody and were adjusted to exclude false positives and false negatives. All images from one experiment were analysed with the same threshold. Results were logged automatically to Microsoft Excel spreadsheets.

For quantification of microglial morphology, the Journal “Microglial Shape” was written in Metamorph. The journal automatically thresholded each image to isolate CD45-positive microglia, then applied the *Integrated Morphometry Analysis* tool *Elliptical Form Factor* (length/breadth) to determine cell shape (Smith et al., 2013b).

### Statistical analysis

Data from representative experiments are displayed, unless otherwise stated, with mean ± standard error of the mean (SEM). Experiments were replicated with cells from at least four different individuals. The F-test and Bartlett’s test were used to check for equal variances. Statistical analysis was carried out using *t*-tests or ANOVA. When no significant interaction was found by two-way ANOVA, one-way ANOVA and Tukey’s multiple comparison tests were used. In cases of unequal variance the equivalent non-parametric test was used (Mann Whitney test or Kruskal-Wallis test with Dunn’s multiple comparison test). *P*-values of < 0.05 were considered statistically significant differences.

## Results

### Differential proliferation of microglia from ventricular/hippocampal and cortical regions

Microglia were cultured from two anatomical regions of biopsy adult human brain tissue from the same patients. Microglia isolated from the cortical middle temporal gyrus were compared to microglia isolated from the neurogenic regions of the hippocampal DG and the overlying SVZ of the lateral ventricle.

**FIGURE 1:**
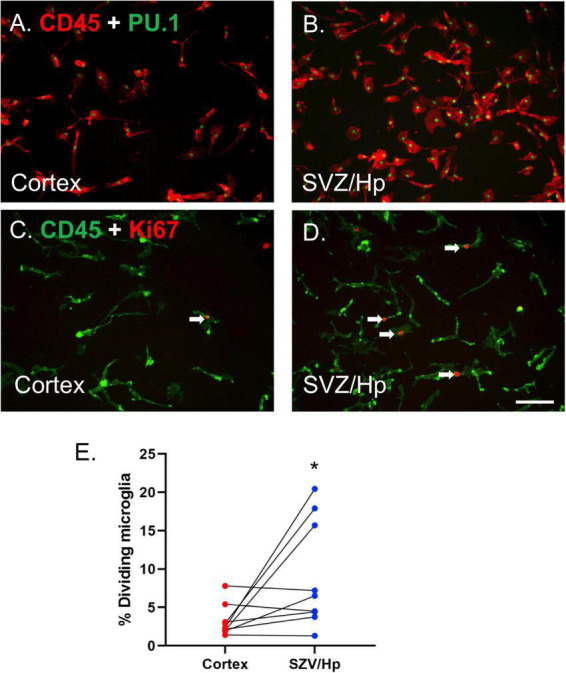
Microglia from neurogenic ventricular/Hp tissue proliferate more than microglia from non-neurogenic cortical tissue. **(A)** PU.1 (green nuclei) and CD45 (red) double-positive microglia are present in glial cultures from cortical human brain tissue. **(B)** A greater number of microglia are present in ventricular/Hp cultures. **(C,D)** Immunocytochemical images of CD45 microglial cell surface marker (green) and Ki67 cell division marker (red) showing minimal proliferation of cortical microglia **(C)** and greater basal proliferation of ventricular/Hp microglia **(D)**. Arrows indicate examples of Ki67-immunopositive microglia. Scale bar = 100 μm. **(E)** Quantification of the percentage of microglia cultured from cortical and ventricular/Hp regions that incorporate BrdU, showing a significantly greater percentage of dividing microglia in ventricular/Hp regions. Each data point indicates an individual case (*n* = 9). Cortex and SVZ/Hp samples from the same case are indicated by connecting lines. *indicates *P* value < 0.05.

Microglia were visualised using antibodies to cell surface antigen CD45 and nuclear transcription factor PU.1 (Smith et al., 2013d; Figures 1A,B). It was found that the yields of microglia (as assessed by immunocytochemistry, Figures 1A,B) from the ventricular/Hp region (580,000 microglia per gram tissue, *n* = 3) were greater than those derived from the cortical middle temporal gyrus region (290,000 microglia per gram tissue, *n* = 3).

Adult human microglia cultured from middle temporal gyrus cortical regions have very low rates of proliferation *in vitro* (3.2 ± 0.7%; *n* = 9), confirming previous results (Gibbons et al., 2007; Smith et al., 2013b). In contrast, microglia derived from the lateral ventricle/Hp areas of the same patients had more variable levels of microglial proliferation, sometimes greatly exceeding that of microglia derived from the cortex. We observed greatly increased microglial proliferation in lateral ventricle/Hp cultures from three out of nine cases; small, marginal increases in another three cases; and no change in three other cases. The endogenous cell division marker Ki67 and the exogenous proliferation indicator BrdU were both used to confirm differences in proliferation and gave equivalent results (Figures 1C–E). Overall, the average basal percentage of dividing microglia in ventricular/Hp cultures was 9.1 ± 2.3% (*n* = 9). The increase in ventricular/Hp microglial division compared to cortical microglial division was variable between cases, but microglia from ventricular/Hp regions were found to have on average a 3-fold (*n* = 9; *p* = 0.040; Mann Whitney test) higher proliferation rate than cortical microglia (Figure 1E).

### Ventricular/hippocampal microglia have a greater proliferation response to Macrophage Colony-Stimulating Factor than cortical microglia

We have previously reported an increase in adult human microglial cell number with M-CSF treatment (Smith et al., 2013b). This increase in microglia cell number was due to increased microglial proliferation. Here we assessed whether microglia cultured from the ventricular/Hp region would also respond to M-CSF by increasing proliferation, given their higher level of basal proliferation.

**FIGURE 2:**
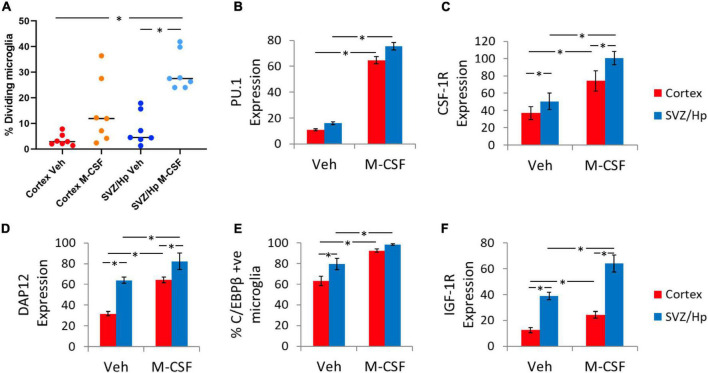
Basal and Macrophage Colony-Stimulating Factor (M-CSF)-induced microglial proliferation and expression of PU.1, CSF-1R, DAP12, CEBPβ, and IGF1 receptor. **(A)** Quantification of the percentage of microglia that incorporate BrdU under control conditions and with M-CSF treatment showing a significant increase in microglial division with M-CSF and an enhanced response in ventricular/Hp microglia. Each data point indicates an individual case (*n* = 7). **(B)** M-CSF significantly increases the intensity (arbitrary fluorescence units) of PU.1 expression (amount of PU.1 protein) in adult human microglia from neurogenic (ventricular/Hp) and non-neurogenic (cortical) regions of the adult human brain. **(C)** CSF-1R is more highly expressed in ventricular/Hp microglia than cortical microglia. A significant increase in intensity of receptor labelling is found for CSF-1R following M-CSF treatment. **(D)** Quantification of DAP12 staining intensity shows a significantly greater level of expression in ventricular/Hp microglia compared to cortical microglia, and a significant increase in DAP12 expression with M-CSF treatment. **(E)** Quantification of microglial C/EBPβ expression showing differential basal expression by cortical and ventricular/Hp microglia, and significant increases in the percentage of cortical and ventricular/Hp microglia which express C/EBPβ following M-CSF treatment. **(F)** Ventricular/Hp microglia express significantly more IGF-1R than cortical microglia, and a significant increase in intensity of IGF-1R is evident following M-CSF treatment. **(B–F)** Protein expression was quantified using an intensity threshold in Metamorph image analysis software, as described in the methods. *indicates *P* value < 0.05.

Macrophage Colony-Stimulating Factor treatment increased proliferation of microglia cultured from the ventricular/Hp region (seven out of seven cases) as for microglia from the cortex. Furthermore, ventricular/Hp microglia had a larger proliferation response to M-CSF than cortical microglia (Figure 2A). At the individual case level, the percentage increase in proliferating microglia with M-CSF treatment was consistently greater for ventricular/Hp microglia than for cortical microglia (five out of seven cases). Although three out of nine cases did not have higher basal ventricular/Hp microglial proliferation compared to cortical microglia, these cases had a greater proliferation response to M-CSF in ventricular/Hp microglia compared to cortical microglia.

### Similar expression of PU.1 transcription factor and CD45 cell surface receptor in ventricular/hippocampal and cortical microglia

PU.1 is a microglial transcription factor which has been shown to be involved in microglial responses to M-CSF (Zhang et al., 1994; Celada et al., 1996; Gómez-Nicola et al., 2013; Smith et al., 2013b). Although there were significantly greater numbers of PU.1-positive microglia in ventricular/Hp cultures, no consistently significant difference was found between cortical and ventricular/Hp microglia in levels of PU.1 protein expression per microglial cell as quantified by the intensity of PU.1 staining (Figure 2B). However, as previously reported for cortical microglia, M-CSF increased the staining intensity of PU.1 indicating an increased amount of PU.1 protein expressed by microglia from both cortical and ventricular/Hp regions (Figure 2B).

It has previously been reported that adult human microglia constitutively express high levels of CD45 *in vitro* (Gibbons et al., 2007; Smith et al., 2013d) and here no difference in CD45 expression was found between ventricular/Hp and cortical microglia (data not shown).

### Neurogenic region microglia express higher levels of proteins involved in Macrophage Colony-Stimulating Factor signalling

To further explore the increased ability of ventricular/Hp microglia to respond to M-CSF, M-CSF receptor (CSF-1R) expression was investigated. We have previously reported the expression of CSF-1R protein on cortical microglia cultured from adult human tissue (Smith et al., 2013b). As expected given their proliferation response to M-CSF, CSF-1R was expressed by microglia cultured from ventricular/Hp as well as cortical regions (Figure 2C). Basal levels of CSF-1R protein expression on cortical and ventricular/Hp microglia were compared and it was found that there was a higher amount of CSF-1R protein expressed by ventricular/Hp microglia than by cortical microglia (Figure 2C). We have previously found that treatment of adult human microglia with M-CSF increases their expression of CSF-1R (Smith et al., 2013b). Both ventricular/Hp and cortical microglia increased expression of CSF-1R following exposure to M-CSF (Figure 2C).

DAP12 is an adaptor protein found in microglia in the adult human brain (Satoh et al., 2011) and is involved in M-CSF signalling (Otero et al., 2009; Smith et al., 2013c). DAP12 expression was assessed in microglia from ventricular/Hp regions and from the cortex. Basal DAP12 expression levels were higher in ventricular/Hp microglia than in cortical microglia for most cases (six out of seven cases; Figure 2D). M-CSF produced an increase in DAP12 expression for cortical microglia (seven out of seven cases; Figure 2D) and this was also observed for ventricular/Hp microglia. Thus ventricular/Hp microglia have higher basal levels of DAP12 expression than cortical microglia and M-CSF treatment of cortical microglia increases their levels of DAP12 expression similar to basal levels in ventricular/Hp microglia (Figure 2D).

C/EBPβ is a transcription factor expressed by microglia which has been shown to be involved in M-CSF-mediated effects (Gómez-Nicola et al., 2013; Smith et al., 2013b). Total microglia were labelled for PU.1 and the percentage of microglia expressing C/EBPβ was quantified (Smith et al., 2013d). Higher C/EBPβ protein expression was found in ventricular/Hp microglia than in cortical microglia (Figure 2E). Furthermore, M-CSF produced an increase in C/EBPβ expression in microglia from both cortex and ventricular/Hp regions (Figure 2E).

Another growth factor that can act on microglia and have immunomodulatory effects is IGF-1. We have previously demonstrated IGF-1R protein expression in human adult microglia (Smith et al., 2013b) and here it was found that basal levels of IGF-1R protein expression are higher in ventricular/Hp microglia than cortical microglia (Figure 2F). We have also previously observed that IGF-1R expression increased on microglia from the cortex following M-CSF treatment. This response was found to also be present in ventricular/Hp microglia (Figure 2F).

### Neurogenic region microglia have a more “activated” phenotype

Two commonly assessed indicators of microglial phenotype are expression of HLA and microglial morphology. We were interested to know whether cortical and ventricular/Hp microglia differed in these measures. HLA is a widely used marker of microglial “activation” and the proportion of microglia expressing HLA basally *in vitro* is highly variable between cases and is further increased by exposure to the pro-inflammatory cytokine IFNy (Smith et al., 2013e). Ventricular/Hp and cortical microglia, from the same cases, were compared to see whether their basal HLA expression differed.

**FIGURE 3:**
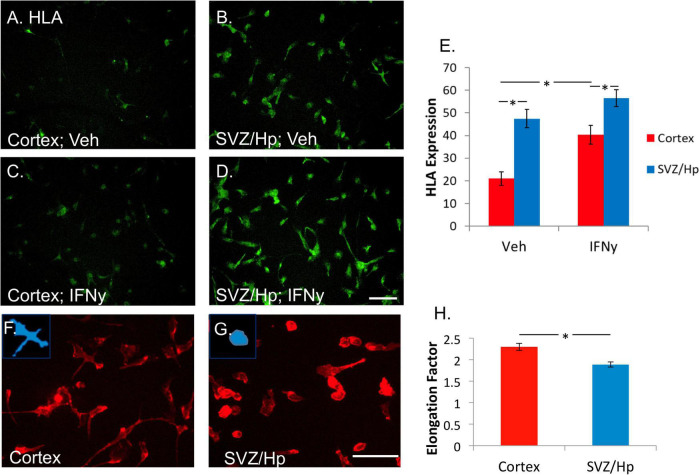
Microglia from the ventricular/Hp region express greater levels of HLA-DP, DQ, and DR are more rounded than cortical microglia. **(A)** In basal conditions without any treatment, a variable level of HLA-DP, DQ, DR is expressed by cortical adult human microglia. **(B)** Microglia from ventricular/Hp regions have a higher basal level of HLA-DP, DQ, DR expression. **(C)** IFNy (1 ng/ml, 96 h) increased cortical microglial expression of HLA-DP, DQ, DR, as well as that of ventricular/Hp microglia **(D)**. Scale bar = 100 μm. **(E)** Quantification of HLA expression (arbitrary fluorescence units) showing differential expression by cortical and ventricular/Hp microglia, and a significant increase in HLA expression with IFNy treatment. **(F)** Adult human microglia isolated from cortical tissue immunolabelled with the cell surface marker CD45 have a heterogeneous morphology with various extended processes. **(G)** Microglia isolated from ventricular/Hp regions have a rounder morphology with fewer processes. Insets, in panels **(F,G)** show representative morphology of cells. Scale bar = 100 μm. **(H)** Quantification of microglial morphology using Metamorph *Elliptical Form Factor* (a measure of elongation) image analysis demonstrates a significant difference in microglia shape between cortical and adjacent neurogenic regions. *indicates *P* value < 0.05.

It was found that ventricular/Hp microglia had a greater propensity to express HLA than cortical microglia. In the majority of cases (six out of seven), more HLA was expressed by ventricular/Hp microglia than cortical microglia (Figures 3A,B,E). IFNy increases cortical adult human microglial expression of HLA (Figure 3C). This was also found to be true for the ventricular/Hp microglia (Figure 3D). However, due to higher basal HLA expression in ventricular/Hp microglia, this effect was not as pronounced as for cortical microglia (Figure 3E).

Human adult microglia are not uniformly shaped *in vitro*. Some are rounded and others have longer processes and extensions. In most of the cases (five out of seven cases) it was observed that microglia cultured from the ventricular/Hp region had rounder morphology than cortical microglia (Figures 3F,G). This difference in cell morphology was quantified and found to be significant (Figure 3H).

A morphological response to M-CSF was observed for both cortical and ventricular/Hp microglia populations. M-CSF caused microglia to become more elongated, confirming previous results (Smith et al., 2013b). The response was found to be quantitatively similar for ventricular/Hp and cortical microglia (data not shown).

## Discussion

Here we report differential proliferation and protein expression of adult human microglia from two distinct brain regions from patients with Mesial Temporal Lobe Epilepsy—(1) the cortex and (2) the hippocampus and overlying ventricular lining.

The initial observation of spontaneously dividing ventricular/Hp microglia in normal culture conditions led to the further investigation of microglia from this region of the adult human brain in comparison to microglia from the cortical temporal lobe. Interestingly, microglia from the ventricular/Hp region were found to proliferate at a relatively high rate without growth factor stimulation, whereas cortical microglia have very low rates of basal proliferation. Using Ki67 immunocytochemistry and BrdU proliferation assays it was demonstrated that the increasing number of ventricular/Hp microglia in culture was due to cell division and not just a survival effect. This finding suggests that there are differences in intrinsic cell division mechanisms in these two microglial populations. Marshall et al. (2008) found that the huge expansive capacity of neonatal rodent SVZ microglia was diminished in the adult brain. The present study shows an increased proliferative capacity of human adult ventricular/Hp microglia, although it is relatively small compared to neonatal rodent microglia. CD45 expression has also previously been shown to be higher in rodent SVZ microglia than microglia in non-neurogenic regions (Goings et al., 2006), however, we did not find this to be the case in adult human microglia.

We investigated whether there was a correlation between microglial proliferation and disease severity, however, no correlation was found between the degree of epilepsy and ventricular/Hp microglial proliferation. From the clinical information available no common factor was evident among the three most highly proliferative cases. This may be due to the small sample size, similar age, and male sex of the patients, and the presence of severe epilepsy in all patients undergoing surgery to treat their symptoms. With larger sample sizes it may be possible to identify sub-populations of individuals with differential microglial proliferation in different brain regions. It is also a possibility that sub-sets of microglia with differential proliferative capacity exist *within* different brain regions, and that more highly proliferative microglia sub-sets were sampled from the three cases with the highest levels of proliferation.

Even more pronounced than the differences in basal proliferation was the difference in M-CSF-stimulated proliferation of ventricular/Hp microglia compared to cortical microglia. The majority of cases exhibited a significantly larger increase in microglial proliferation with M-CSF for the ventricular/Hp region compared to the cortex. M-CSF is present in the adult human brain and has been reported to be differentially expressed in varying disease states, thus this growth factor may stimulate microglial proliferation in the intact human brain.

Based on our previous findings with adult human microglia and characterisation of their responses to M-CSF (Smith et al., 2013b), we used a panel of microglial proteins to compare microglia isolated from the cortex and ventricular/Hp regions. To identify the mechanisms behind this differential proliferative response in microglia from two distinct brain regions, expression of the receptor for M-CSF (CSF-1R) was assessed. In concordance with their heightened response to M-CSF, higher expression of CSF-1R protein was found on ventricular/Hp microglia. Higher CSF-1R expression by ventricular/Hp microglia was not paralleled by relatively high PU.1 expression, even though PU.1 has been shown to regulate CSF-1R gene expression (Zhang et al., 1994). However, greater expression of the C/EBPβ transcription factor in ventricular/Hp microglia could be mechanistically involved in their increased response to M-CSF. C/EBPβ has been shown to regulate CSF-1R gene expression (Zhang et al., 1996) and to be involved in M-CSF actions in disease states (Komuro et al., 2003; Marigo et al., 2010). Increased DAP12 expression in ventricular/Hp microglia could also be related to their increased response to M-CSF as we have previously shown that M-CSF treatment increases DAP12 expression in adult human microglia (Smith et al., 2013b), and M-CSF has been found to induce macrophage proliferation *via* DAP12 (Otero et al., 2009). Thus the machinery for M-CSF signalling—CSF-1R, DAP12 adaptor protein and C/EBPβ transcription factor—are more highly expressed in ventricular/Hp microglia, associated with a strong proliferative response to M-CSF.

The mitogenic growth factor IGF-1 may share some functional effects with M-CSF (Wessells et al., 2004; Gow et al., 2010) and has also been reported to be expressed in the adult brain (Connor et al., 1997). Here it was found that the receptor for IGF-1 (IGF-1R) was also expressed at higher basal levels in ventricular/Hp microglia compared to cortical microglia. The finding that protein levels of IGF-1R are increased upon M-CSF stimulation confirms our previous reports with adult human microglia (Smith et al., 2013b). IGF-1 has been reported within neurogenic regions (Anderson et al., 2002). Given the important role that IGF-1 is thought to play in adult neurogenesis, this intriguing finding of increased IGF-1R expression on ventricular/Hp microglia shows that IGF-1 may act through microglia, as well as other cell types including NPCs (Aberg et al., 2003; Monzo et al., 2013), in neurogenic regions to influence neurogenesis.

Regional differences in the response of rodent microglia to cytokine receptor-stimulation have previously been reported, and together these findings raise the question of whether there are truly different “sub-populations” of microglia in different brain regions or whether all microglia will respond similarly if placed in the same environment (van Weering et al., 2011; Hanisch, 2013). The results from this study show that microglia from different brain regions retain differential phenotype and function *in vitro*, indicating some level of autonomous cell phenotype, but to what extent these microglial phenotypes are reversible *in vivo* is still unknown.

Functional microglial diversity in specific brain regions is likely necessary to accommodate the requirements of different brain regions, for example different energy requirements and cell types. The observation of a distinct profile of microglia from the ventricular lining and Hp, which are adult neurogenic regions, is intriguing as adult neurogenesis requires immune support (Martino et al., 2011; Morrens et al., 2012) but exactly how the finding of increased proliferation of microglia in stem cell niches has an effect on the NPCs residing there is unknown. NPCs and microglia both release trophic and immunomodulatory molecules (Pluchino et al., 2005; Martino and Pluchino, 2007). In fact NPCs are being discovered to have remarkable influence over immune activity and were found to have a distinct secretory protein profile (Mosher et al., 2012). Conditioned medium from primary mouse NPCs was found to induce microglial proliferation, chemotaxis and phagocytosis, while transplantation of NPCs or NPC conditioned medium significantly increased the numbers of dividing microglia *in vivo* (Mosher et al., 2012). Thus it seems that microglia and NPC have a two-way relationship, both contributing to maintenance of the neurogenic niche.

The rounder morphology and higher levels of HLA protein expression of ventricular/Hp microglia compared to their cortical counterparts are generally thought to be indicative of an “activated, pro-inflammatory” microglial phenotype (Graeber, 2010). With a concurrent increase in proliferation, this may identify an important microglia phenotype or sub-type with disease-relevant implications for neurogenic regions in Mesial Temporal Lobe Epilepsy. Furthermore, it is possible that an inflammatory environment resulting from seizure activity is responsible for the increased proliferation and activation of neurogenic region microglia in Mesial Temporal Lobe Epilepsy tissue. Further studies are required to address whether increased proliferation combined with high HLA expression results in a harmful or beneficial microglia phenotype in ventricular/Hp regions, both in patients with Mesial Temporal Lobe Epilepsy and in healthy controls. Specifically, assaying pro-inflammatory cytokine secretion from neurogenic region and cortical region microglia would advance our understanding of the functional implications of the current findings.

Conversely, adult human microglial responses to M-CSF have been shown to result in microglia with “surveying” characteristics. M-CSF induces a major morphological response in adult human microglia whereby they become elongated and bipolar (Smith et al., 2013b). Furthermore, M-CSF has been shown to reduce microglial HLA expression, including that of primary adult human microglia. However, microglia respond to many factors in their environment simultaneously and the phenotypic plasticity of microglia is highly evident here, where ventricular/Hp microglia are shown to be basally “activated” but have massive responses to M-CSF through proliferation, morphology change and multiple protein expression changes.

Our findings are in-line with a recent study of *post-mortem* human microglia which found that SVZ microglia had significantly higher expression of HLA-DR as well as CD45, CD68 (a lysosomal protein), and CD64 (Fc receptor), compared to microglia from the temporal and frontal cortex, by FACS and CyTOF (Böttcher et al., 2019). They also noted that these microglia expressed higher levels of proliferation markers (Böttcher et al., 2019). This suggests that our findings are indeed representative of region-specific differences in microglia and not an artefact of biopsy tissue.

A possible explanation for heightened ventricular/Hp microglial activation is their proximity to immune protein-containing cerebrospinal fluid in the lateral ventricles. It has been demonstrated that microglia in regions of the brain with a less defined blood-brain barrier, and thus increased exposure to plasma proteins, have a less ramified morphology than microglia from other regions (Cuadros and Navascues, 1998; Galea et al., 2007). However, we do not yet know whether the *in vitro* differences in morphology observed here are also present in human brains, or in 3D *in vitro* cultures.

This study utilises biopsy human brain tissues which are invaluable for understanding adult human brain disorders but which undoubtedly come with several limitations. As biopsy brain tissue cannot be taken from healthy individuals, this study is limited to tissue from chronically diseased individuals. It is unknown to what extent the disease process has led to the observations in this study, and whether the same holds true for healthy human brain tissue. Furthermore, although the seizure focus was removed by a pathologist before separation of cortical and ventricular/Hp tissue and not used for microglia isolation, an alternative explanation for the microglial differences observed in these two brain regions could be their relative distance from the seizure focal point, as even the temporal cortex microglia may be influenced by seizure activity in the neighbouring hippocampus, or a response to ongoing neurodegeneration in the hippocampal area as part of epilepsy pathology. Despite these limitations, use of this tissue forms an important part of our understanding of the role of microglia in the adult human brain.

In conclusion, we report fundamental differences in two regional populations of microglia in the adult human brain. Specifically, we observed higher rates of proliferation in neurogenic region microglia and a heightened proliferative response to the mitogen M-CSF. Furthermore, neurogenic region microglia display characteristics of activation. The functional differences among microglia based on regional variation adds to the emerging view of microglia as a highly heterogeneous and phenotypically diverse cell type and may have relevance to the maintenance of the neurogenic niche in the adult human brain.

## Data availability statement

The original contributions presented in this study are included in the article/Supplementary material, further inquiries can be directed to the corresponding authors.

## Ethics statement

The studies involving human participants were reviewed and approved by University of Auckland Human Participants Ethics Committee and Northern Regional Ethics Committee. The patients/participants provided their written informed consent to participate in this study.

## Author contributions

AS and MD conceived and designed the experiments, interpreted the data, and wrote the manuscript. AS performed the cell isolation, cell culture, immunocytochemistry, image acquisition, and analysis. TP performed the isolation and culture of cells. MA performed the immunocytochemistry. RO, PB, EM, and RF contributed to materials and clinical information and revised the manuscript. All authors read and approved the final manuscript.

## Supplementary material

Supplementary Figure 1 Blocking of non-specific antibody binding to Fc receptors did not alter staining patterns. Following fixation, cells were incubated with PBS or purified recombinant Fc protein to block non-specific binding of antibodies to the Fc receptor. Staining was then performed using the indicated primary and appropriate secondary antibodies at the concentrations used for all other experiments. Representative images are shown. Scale bar = 100 μm.

## References

[B1] Aberg M. A. Aberg N. D. Palmer T. D. Alborn A. M. Carlsson-Skwirut C. Bang P. (2003). Igf-I has a direct proliferative effect in adult hippocampal progenitor cells. *Mol. Cell. Neurosci.* 24 23–40. 10.1016/S1044-7431(03)00082-4 14550766

[B2] Anderson M. F. Aberg M. A. I. Nilsson M. Eriksson P. S. (2002). Insulin-like growth factor-I and neurogenesis in the adult mammalian brain. *Dev. Brain Res.* 134 115–122. 10.1016/S0165-3806(02)00277-8 11947942

[B3] Böttcher C. Schlickeiser S. Sneeboer M. A. M. Kunkel D. Knop A. Paza E. (2019). Human microglia regional heterogeneity and phenotypes determined by multiplexed single-cell mass cytometry. *Nat. Neurosci.* 22 78–90. 10.1038/s41593-018-0290-2 30559476

[B4] Celada A. Borras F. Soler C. Lloberas J. Klemsz M. Van Beveren C. (1996). The transcription factor Pu.1 is involved in macrophage proliferation. *J. Exp. Med.* 184 61–69. 10.1084/jem.184.1.61 8691150 PMC2192661

[B5] Chan A. Seguin R. Magnus T. Papadimitriou C. Toyka K. Antel J. (2001). Phagocytosis of apoptotic inflammatory cells by microglia is modulated by cytokines. *Glia* 33 87–95. 10.1002/1098-1136(20010101)33:1<87::AID-GLIA1008>3.0.CO;2-S 11169794

[B6] Connor B. Beilharz E. J. Williams C. Synek B. Gluckman P. D. Faull R. L. (1997). Insulin-like growth factor-I (Igf-I) immunoreactivity in the Alzheimer’s disease temporal cortex and hippocampus. *Brain Res. Mol. Brain Res.* 49 283–290. 10.1016/S0169-328X(97)00192-7 9387889

[B7] Cuadros M. A. Navascues J. (1998). The origin and differentiation of microglial cells during development. *Prog. Neurobiol.* 56 173–189. 10.1016/S0301-0082(98)00035-5 9760700

[B8] Curtis M. A. Kam M. Nannmark U. Anderson M. F. Axell M. Z. Wikkelso C. (2007). human neuroblasts migrate to the olfactory bulb via a lateral ventricular extension. *Science* 315 1243–1249. 10.1126/science.1136281 17303719

[B9] Curtis M. A. Waldvogel H. J. Synek B. Faull R. L. M. (2005). A histochemical and immunohistochemical analysis of the subependymal layer in the normal and Huntington’s disease brain. *J. Chem. Neuroanat.* 30 55–66. 10.1016/j.jchemneu.2005.05.001 16108100

[B10] de Haas A. H. Boddeke H. W. G. M. Biber K. (2008). Region-specific expression of immunoregulatory proteins on microglia in the healthy Cns. *Glia* 56 888–894. 10.1002/glia.20663 18338796

[B11] de Haas A. H. Van Weering H. R. De Jong E. K. Boddeke H. W. Biber K. P. (2007). Neuronal chemokines: Versatile messengers in central nervous system cell interaction. *Mol. Neurobiol.* 36 137–151. 10.1007/s12035-007-0036-8 17952658 PMC2039784

[B12] Dragunow M. (2008a). The adult human brain in preclinical drug development. *Nat. Rev.* 7 659–666. 10.1038/nrd2617 18617887

[B13] Dragunow M. (2008b). High-content analysis in neuroscience. *Nat. Rev. Neurosci.* 9 779–788. 10.1038/nrn2492 18784656

[B14] Eriksson P. S. Perfilieva E. Bjork-Eriksson T. Alborn A.-M. Nordborg C. Peterson D. A. (1998). Neurogenesis in the adult human hippocampus. *Nat. Med.* 4 1313–1317. 10.1038/3305 9809557

[B15] Galea I. Bechmann I. Perry V. H. (2007). What is immune privilege (not)? *Trends Immunol.* 28 12–18. 10.1016/j.it.2006.11.004 17129764

[B16] Gibbons H. M. Dragunow M. (2010). Adult human brain cell culture for neuroscience research. *Int. J. Biochem. Cell Biol.* 42 844–856. 10.1016/j.biocel.2009.12.002 20004737

[B17] Gibbons H. M. Hughes S. M. Van Roon-Mom W. Greenwood J. M. Narayan P. J. Teoh H. H. (2007). Cellular composition of human glial cultures from adult biopsy brain tissue. *J. Neurosci. Methods* 166 89–98. 10.1016/j.jneumeth.2007.07.005 17719090

[B18] Gibbons H. M. Smith A. M. Teoh H. H. Bergin P. M. Mee E. W. Faull R. L. M. (2011). Valproic acid induces microglial dysfunction, not apoptosis, in human glial cultures. *Neurobiol. Dis.* 41 96–103. 10.1016/j.nbd.2010.08.024 20816784

[B19] Ginhoux F. Greter M. Leboeuf M. Nandi S. See P. Gokhan S. (2010). Fate mapping analysis reveals that adult microglia derive from primitive macrophages. *Science* 330 841–845. 10.1126/science.1194637 20966214 PMC3719181

[B20] Goings G. E. Kozlowski D. A. Szele F. G. (2006). Differential activation of microglia in neurogenic versus non-neurogenic regions of the forebrain. *Glia* 54 329–342. 10.1002/glia.20381 16862532

[B21] Gómez-Nicola D. Fransen N. Suzzi S. Perry V. (2013). Regulation of microglial proliferation during chronic neurodegeneration. *J. Neurosci.* 33 2481–2493. 10.1523/JNEUROSCI.4440-12.2013 23392676 PMC6619184

[B22] Gow D. J. Sester D. P. Hume D. A. (2010). Csf-1, Igf-1, and the control of postnatal growth and development. *J. Leukocyte Biol.* 88 475–481. 10.1189/jlb.0310158 20519640

[B23] Graeber M. B. (2010). Changing face of microglia. *Science* 330 783–788. 10.1126/science.1190929 21051630

[B24] Hanisch U.-K. (2013). Functional diversity of microglia – how heterogeneous are they to begin with? *Front. Cell. Neurosci.* 7:65. 10.3389/fncel.2013.00065 23717262 PMC3653062

[B25] Hart A. D. Wyttenbach A. Hugh Perry V. Teeling J. L. (2012). Age related changes in microglial phenotype vary between Cns regions: Grey versus white matter differences. *Brain Behav. Immun.* 26 754–765. 10.1016/j.bbi.2011.11.006 22155499 PMC3381227

[B26] Hellstrom N. A. K. Lindberg O. R. Stahlberg A. Swanpalmer J. Pekny M. Blomgren K. (2011). Unique gene expression patterns indicate microglial contribution to neural stem cell recovery following irradiation. *Mol. Cell. Neurosci.* 46 710–719. 10.1016/j.mcn.2011.02.001 21315821

[B27] Komuro I. Yokota Y. Yasuda S. Iwamoto A. Kagawa K. S. (2003). Csf-induced and Hiv-1-mediated distinct regulation of Hck and C/Ebpbeta represent a heterogeneous susceptibility of monocyte-derived macrophages to M-tropic Hiv-1 Infection. *J. Exp. Med.* 198 443–453. 10.1084/jem.20022018 12900520 PMC2194092

[B28] Lee S. C. Liu W. Roth P. Dickson D. W. Berman J. W. Brosnan C. F. (1993). Macrophage colony-stimulating factor in human fetal astrocytes and microglia. Differential regulation by cytokines and lipopolysaccharide, and modulation of class Ii Mhc on microglia. *J. Immunol.* 150 594–604. 8419491

[B29] Marigo I. Bosio E. Solito S. Mesa C. Fernandez A. Dolcetti L. (2010). Tumor-induced tolerance and immune suppression depend on the C/Ebpbeta transcription factor. *Immunity* 32 790–802. 10.1016/j.immuni.2010.05.010 20605485

[B30] Marshall G. P. II Deleyrolle L. P. Reynolds B. A. Steindler D. A. Laywell E. D. (2014). Microglia from neurogenic and non-neurogenic regions display differential proliferative potential and neuroblast support. *Front. Cell. Neurosci.* 8:180. 10.3389/fncel.2014.00180 25076873 PMC4100441

[B31] Marshall G. P. Demir M. Steindler D. A. Laywell E. D. (2008). Subventricular zone microglia possess a unique capacity for massive in vitro expansion. *Glia* 56 1799–1808. 10.1002/glia.20730 18661554 PMC4424978

[B32] Martino G. Pluchino S. (2007). Neural stem cells: Guardians of the brain. *Nat. Cell. Biol.* 9 1031–1034. 10.1038/ncb0907-1031 17762897

[B33] Martino G. Pluchino S. Bonfanti L. Schwartz M. (2011). Brain regeneration in physiology and pathology: The immune signature driving therapeutic plasticity of neural stem cells. *Physiol. Rev.* 91 1281–1304. 10.1152/physrev.00032.2010 22013212 PMC3552310

[B34] Melief J. Koning N. Schuurman K. G. Van De Garde M. D. B. Smolders J. Hoek R. M. (2012). Phenotyping primary human microglia: Tight regulation of Lps responsiveness. *Glia* 60 1506–1517. 10.1002/glia.22370 22740309

[B35] Mittelbronn M. Dietz K. Schluesener H. J. Meyermann R. (2001). Local distribution of microglia in the normal adult human central nervous system differs by up to one order of magnitude. *Acta Neuropathol.* 101 249–255. 10.1007/s004010000284 11307625

[B36] Monzo H. J. Park T. I. H. Dieriks V. B. Jansson D. Faull R. L. M. Dragunow M. (2013). Insulin and Igf1 modulate turnover of polysialylated neuronal cell adhesion molecule (Psa-Ncam) in a process involving specific extracellular matrix components. *J. Neurochem.* 126 758–770. 10.1111/jnc.12363 23844825

[B37] Morrens J. Van Den Broeck W. Kempermann G. (2012). Glial cells in adult neurogenesis. *Glia* 60 159–174. 10.1002/glia.21247 22076934

[B38] Mosher K. I. Andres R. H. Fukuhara T. Bieri G. Hasegawa-Moriyama M. He Y. (2012). Neural progenitor cells regulate microglia functions and activity. *Nat. Neurosci.* 15 1485–1487. 10.1038/nn.3233 23086334 PMC3495979

[B39] Nimmerjahn A. Kirchhoff F. Helmchen F. (2005). Resting microglial cells are highly dynamic surveillants of brain parenchyma in vivo. *Science* 308 1314–1318. 10.1126/science.1110647 15831717

[B40] Olah M. Biber K. Vinet J. Boddeke H. (2011). Microglia phenotype diversity. *CNS Neurol. Disorder. Drug Targets* 10 108–118. 10.2174/187152711794488575 21143141

[B41] Otero K. Turnbull I. R. Poliani P. L. Vermi W. Cerutti E. Aoshi T. (2009). Macrophage colony-stimulating factor induces the proliferation and survival of macrophages via a pathway involving Dap12 and [beta]-catenin. *Nat. Immunol.* 10 734–743. 10.1038/ni.1744 19503107 PMC4004764

[B42] Park T. I. Smyth L. C. D. Aalderink M. Woolf Z. R. Rustenhoven J. Lee K. (2022). Routine culture and study of adult human brain cells from neurosurgical specimens. *Nat. Protoc.* 17 190–221. 10.1038/s41596-021-00637-8 35022619

[B43] Park T. I.-H. Monzo H. Mee E. W. Bergin P. S. Teoh H. H. Montgomery J. M. (2012). Adult human brain neural progenitor cells (Npcs) and fibroblast-like cells have similar properties in vitro but only Npcs differentiate into neurons. *PLoS One* 7:e37742. 10.1371/journal.pone.0037742 22675489 PMC3366988

[B44] Pluchino S. Zanotti L. Rossi B. Brambilla E. Ottoboni L. Salani G. (2005). Neurosphere-derived multipotent precursors promote neuroprotection by an immunomodulatory mechanism. *Nature* 436 266–271. 10.1038/nature03889 16015332

[B45] Rustenhoven J. Park T. I. H. Schweder P. Scotter J. Correia J. Smith A. M. (2016). Isolation of highly enriched primary human microglia for functional studies. *Sci. Rep.* 6:19371. 10.1038/srep19371 26778406 PMC4725991

[B46] Satoh J.-I. Tabunoki H. Ishida T. Yagishita S. Jinnai K. Futamura N. (2011). Immunohistochemical characterization of microglia in Nasu-Hakola disease brains. *Neuropathology* 31 363–375. 10.1111/j.1440-1789.2010.01174.x 21118401

[B47] Sierra A. Encinas J. M. Deudero J. J. P. Chancey J. H. Enikolopov G. Overstreet-Wadiche L. S. (2010). Microglia shape adult hippocampal neurogenesis through apoptosis-coupled phagocytosis. *Cell Stem Cell* 7 483–495. 10.1016/j.stem.2010.08.014 20887954 PMC4008496

[B48] Simard A. R. Soulet D. Gowing G. Julien J.-P. Rivest S. (2006). Bone marrow-derived microglia play a critical role in restricting senile plaque formation in Alzheimer’s Disease. *Neuron* 49 489–502. 10.1016/j.neuron.2006.01.022 16476660

[B49] Smith A. M. Dragunow M. (2014). The human side of microglia. *Trends Neurosci.* 37 125–135. 10.1016/j.tins.2013.12.001 24388427

[B50] Smith A. M. Gibbons H. M. Dragunow M. (2010). Valproic acid enhances microglial phagocytosis of amyloid-b1-42. *Neuroscience* 169 505–515. 10.1016/j.neuroscience.2010.04.041 20423723

[B51] Smith A. Gibbons H. Lill C. Faull R. M. Dragunow M. (2013a). “Isolation and culture of adult human microglia within mixed glial cultures for functional experimentation and high-content analysis,” in *Microglia*, eds Joseph B. Venero J. L. (Totowa, NJ: Humana Press). 10.1007/978-1-62703-520-0_6 23813368

[B52] Smith A. Gibbons H. Oldfield R. Bergin P. Mee E. Curtis M. (2013b). M-Csf increases proliferation and phagocytosis while modulating receptor and transcription factor expression in adult human microglia. *J. Neuroinflammation* 10:85. 23866312 10.1186/1742-2094-10-85 PMC3729740

[B53] Smith A. M. Gibbons H. M. Oldfield R. L. Bergin P. M. Mee E. W. Faull R. L. M. (2013d). The transcription factor Pu.1 is critical for viability and function of human brain microglia. *Glia* 61 929–942. 10.1002/glia.22486 23483680

[B54] Smith A. M. Gibbons H. M. Oldfield R. L. Bergin P. M. Mee E. W. Curtis M. A. (2013c). M-Csf increases proliferation and phagocytosis while modulating receptor and transcription factor expression in adult human microglia. *J. Neuroinflammation* 10:85. 10.1186/1742-2094-10-85 23866312 PMC3729740

[B55] Smith A. M. Graham E. S. Feng S. X. Oldfield R. L. Bergin P. M. Mee E. W. (2013e). Adult human glia, pericytes and meningeal fibroblasts respond similarly to Ifny but not to Tgfbeta1 or M-Csf. *PLoS One* 8:e80463. 10.1371/journal.pone.0080463 24339874 PMC3855168

[B56] van Weering H. R. J. Boddeke H. W. G. M. Vinet J. Brouwer N. De Haas A. H. Van Rooijen N. (2011). Cxcl10/Cxcr3 signaling in glia cells differentially affects Nmda-induced cell death in Ca and Dg neurons of the mouse hippocampus. *Hippocampus* 21 220–232. 10.1002/hipo.20742 20082289

[B57] Vidyadaran S. Ooi Y. Y. Subramaiam H. Badiei A. Abdullah M. Ramasamy R. (2009). Effects of macrophage colony-stimulating factor on microglial responses to lipopolysaccharide and beta amyloid. *Cell. Immunol.* 259 105–110. 10.1016/j.cellimm.2009.06.005 19577228

[B58] Wake H. Moorhouse A. Jinno S. Kohsaka S. Nabekura J. (2009). Resting microglia directly monitor the functional state of synapses in vivo and determine the fate of ischemic terminals. *J. Neurosci.* 29 3974–3980. 10.1523/JNEUROSCI.4363-08.2009 19339593 PMC6665392

[B59] Walton N. M. Sutter B. M. Laywell E. D. Levkoff L. H. Kearns S. M. Marshall G. P. (2006). Microglia instruct subventricular zone neurogenesis. *Glia* 54 815–825. 10.1002/glia.20419 16977605

[B60] Wessells J. Yakar S. Johnson P. F. (2004). Critical prosurvival roles for C/Ebpbeta and insulin-like growth factor I in macrophage tumor cells. *Mol. Cell. Biol.* 24 3238–3250. 10.1128/MCB.24.8.3238-3250.2004 15060147 PMC381667

[B61] Yamamoto S. Nakajima K. Kohsaka S. (2010). Macrophage-colony stimulating factor as an inducer of microglial proliferation in axotomized rat facial nucleus. *J. Neurochem.* 115 1057–1067. 10.1111/j.1471-4159.2010.06996.x 20831658

[B62] Zhang D. E. Hetherington C. J. Chen H. M. Tenen D. G. (1994). The macrophage transcription factor Pu.1 directs tissue-specific expression of the macrophage-colony-stimulating factor receptor. *Mol. Cell. Biol.* 14 373–381. 10.1128/mcb.14.1.373-381.1994 8264604 PMC358386

[B63] Zhang D. E. Hetherington C. J. Meyers S. Rhoades K. L. Larson C. J. Chen H. M. (1996). Ccaat enhancer-binding protein (C/Ebp) and Aml1 (Cbf alpha 2) synergistically activate the macrophage colony-stimulating factor receptor promoter. *Mol. Cell. Biol.* 16 1231–1240. 10.1128/MCB.16.3.1231 8622667 PMC231105

