# Supplementary figures and images for: Distinct characteristics of microglia from neurogenic and non-neurogenic regions of the human brain in patients with Mesial Temporal Lobe Epilepsy
Source: Front Cell Neurosci. 2022 Nov 8;16:1047928. doi: 10.3389/fncel.2022.1047928 (PMC9679155; doi:10.3389/fncel.2022.1047928)

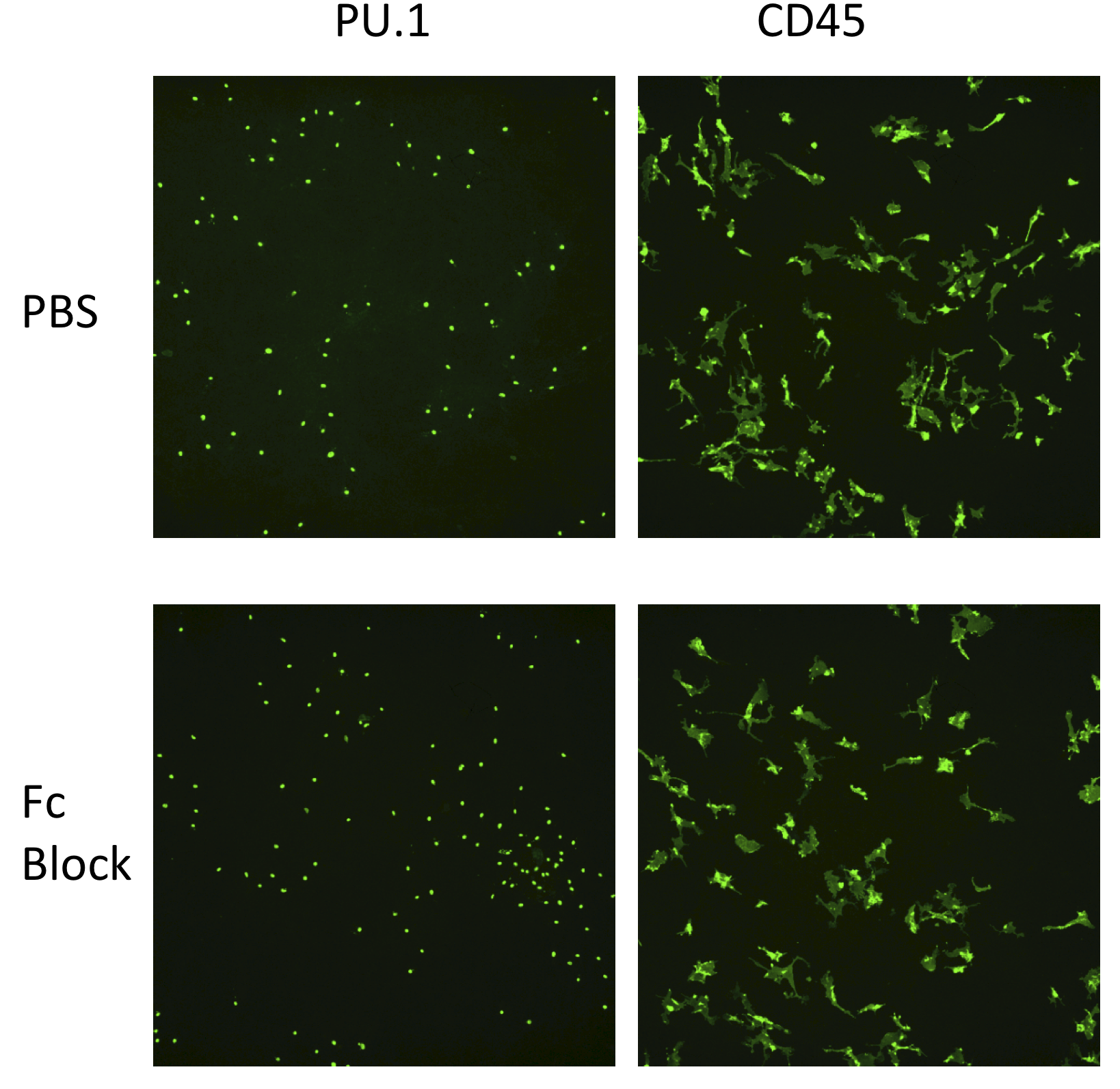

Supplement: Supplementary Figure 1 — Blocking of non-specific antibody binding to Fc receptors did not alter staining patterns. Following fixation, cells were incubated with PBS or purified recombinant Fc protein to block non-specific binding of antibodies to the Fc receptor. Staining was then performed using the indicated primary and appropriate secondary antibodies at the concentrations used for all other experiments. Representative images are shown. Scale bar = 100 μm. [file Image_1.tiff]
